# An Exploratory Study of Shopping to Relieve Tension or Anxiety in Adolescents: Health Correlates and Gambling-Related Perceptions and Behaviors

**DOI:** 10.3390/ijerph19106169

**Published:** 2022-05-19

**Authors:** Luis C. Farhat, Zu Wei Zhai, Rani A. Hoff, Suchitra Krishnan-Sarin, Marc N. Potenza

**Affiliations:** 1 Department of Psychiatry, Faculdade de Medicina FMUSP, Universidade de Sao Paulo, Sao Paulo 05403-903, Brazil; luis.farhat@usp.br; 2 Program in Neuroscience, Middlebury College, Middlebury, VT 05753, USA; zuweizhai@gmail.com; 3 Department of Psychiatry, Yale School of Medicine, New Haven, CT 06510, USA; rani.hoff@va.gov (R.A.H.); suchitra.krishnan-sarin@yale.edu (S.K.-S.); 4 Northeast Program Evaluation Center, VA Connecticut Healthcare System, West Haven, CT 06516, USA; 5 Connecticut Mental Health Center, New Haven, CT 06519, USA; 6 Child Study Center, Yale School of Medicine, New Haven, CT 06511, USA; 7 Department of Neuroscience, Yale School of Medicine, New Haven, CT 06510, USA; 8 Connecticut Council on Problem Gambling, Wethersfield, CT 06109, USA; 9 Wu Tsai Institute, Yale University, New Haven, CT 06510, USA

**Keywords:** epidemiologic studies, addictive behaviors, adolescent, gambling, compulsive behavior, shopping, anxiety, substance use

## Abstract

The desire to escape from pressures/anxiety represents an important motivation for problematic engagement with short-term rewarding behaviors that could contribute to the development of recognized and candidate behavioral addictions, including problematic shopping, a prevalent condition among youth in the U.S.A. characterized by excessive shopping cognitions/behaviors that lead to distress/impairment. However, to date, the specific correlates of shopping to relieve anxiety or tension have yet to be evaluated. We aimed at addressing this gap by analyzing data (N = 2556) from a high-school survey from Connecticut in an exploratory fashion. Adolescents who acknowledged experiencing a growing tension or anxiety that could only be relieved by shopping were classified as having negative-reinforcement shopping and compared to the remaining students. Data were analyzed in chi-square and logistic regression models to examine negative-reinforcement shopping in relation to socio-demographics, health correlates, gambling-related perceptions/attitudes, and problem-gambling severity/gambling behaviors. Negative-reinforcement shopping was more frequent in female and Hispanic students, was linked to more permissive gambling attitudes and at-risk/problematic gambling, and was associated with the use of alcohol, tobacco, marijuana, and other drugs, dysphoria/depression, and weapon-carrying and physical fighting. Taken together, these findings highlight adverse measures of health and functioning linked to negative-reinforcement shopping that may be particularly relevant to girls and Hispanic youth. Additional efforts to prevent negative outcomes are warranted.

## 1. Introduction

### 1.1. Behavioral Addictions

The term ‘behavioral addictions’ has been used to describe patterns of engagement with short-term rewarding activities other than psychoactive-substance use which are characterized by core elements of addiction (e.g., poor control, sustained engagement despite adverse consequences, cravings/urges before engagement) [1,2]. Although there exists some debate regarding what conditions should be classified as behavioral addictions [3,4], clinical researchers have long noticed [5] that some individuals experience negative personal, social, academic/occupational, physical and/or mental health consequences due to problematic engagement in behaviors such as buying [6], gambling [7], gaming [8] and working [9], among others. Therefore, additional research on recognized and candidate behavioral addictions is important to understand these potentially impairing conditions and contribute to the advancement of the current state of knowledge.

A potentially fruitful approach in the investigation of recognized and candidate behavioral addictions is to consider underlying motivations for the engagement in the related short-term rewarding behaviors. Motivational characteristics are important components of theoretical models of addiction [10,11], and empirical evidence from animal models [12] and neuroimaging research [13] has supported the importance of positive/negative reinforcement in the development of addictions. In the context of behavioral addictions, positive-reinforcement motivations such as sensation-/excitement-seeking have been explored in previous research, for example, with gambling [14,15,16]; however, negative reinforcement mechanisms such as being motivated by a desire to relieve tension or anxiety have been less well studied [17]. Thus, additional research on the topic is currently required.

### 1.2. Problematic Shopping

Problematic shopping (PS) is a candidate behavioral addiction characterized by excessive shopping cognitions and buying behaviors that lead to distress and/or impairment [6]. PS is common among adults and adolescents in the U.S. with prevalence estimates of 5.8% and 3.5%, respectively [18,19]. Treatment-seeking samples have suggested females are considerably more likely to engage in PS [20,21], but data from large-scale representative surveys of the general population indicate that PS has a relatively balanced gender distribution [18,19].

PS during adolescence is of particular concern because it has been associated with several negative health correlates such as substance use (e.g., tobacco, marijuana, and other synthetic drugs), depression, antisocial behaviors (e.g., fighting, carrying weapons), and engagement in non-suicidal self-injury [19,22,23]. Notably, most adults with PS report age-at-onset of their behavior during adolescence [24], suggesting their behavior may begin early in development and persist across the lifespan. Therefore, proper identification of PS behaviors at an early age during adolescence could possibly help in developing prevention strategies for PS behaviors across the lifespan.

### 1.3. Negative-Reinforcement Shopping

Conceptual models [25,26] and empirical evidence [27,28,29] suggest that the desire to escape from pressures/anxiety is an important motivation underlying PS. Negative-reinforcement engagement in shopping could precede the development of PS, and the identification of characteristics associated with engagement in shopping to relieve anxiety/tension could help in the recognition and prevention of PS, as has been suggested for behavioral addictions more generally [30,31]. However, to date, the specific correlates (e.g., socio-demographic and health characteristics) of negative-reinforcement shopping are not well understood, particularly among youth.

Available evidence suggests that individuals who shop to relieve anxiety/tension could be at risk of engagement in other addictive behaviors, e.g., substance use and problem gambling. In a previous study of the data from the high-school survey on which the current analyses are based, adolescents with at-risk/problem gambling were more likely to report negative-reinforcement shopping [32], suggesting that shopping to relieve tension/anxiety could be particularly associated with at-risk/problem gambling. Indeed, it is possible that adolescents may engage in multiple short-term rewarding activities to relieve tension/anxiety (e.g., gambling, substance use). However, to date, substance-use and gambling correlates of shopping to relieve tension/anxiety have not been examined among youth.

### 1.4. Study Approach and Hypotheses

This study aims at addressing gaps in knowledge by evaluating, in an exploratory fashion, socio-demographics, health measures, gambling perceptions and problem-gambling severity/gambling behaviors in adolescent high-school students from Connecticut stratified by negative-reinforcement shopping status. We hypothesized that negative-reinforcement shopping, operationalized as shopping to relieve tension or anxiety, would be associated with substance use, dysphoria/depression, and aggressive/violent behaviors; more permissive attitudes towards gambling; and at-risk/problem gambling. We also explored different preferred gambling locations (e.g., casinos) and types of gambling (e.g., non-strategic) in relation to negative-reinforcement shopping.

## 2. Materials and Methods

### 2.1. Recruitment and Sample Characteristics

Data were drawn from a cross-sectional survey of high-school students from the state of Connecticut. The recruitment and data-collection procedures have been previously described in detail in other publications [33,34]. Briefly, all public four-year non-vocational or special-education high-schools in the state of Connecticut were invited to participate in the survey via invitation letters and follow-up phone calls during 2006. Targeted recruitment was subsequently conducted to ensure representation of originally underrepresented regions. The final sample demographics were consistent with reports of the 2000 Census of Connecticut residents between 14 and 18 years of age.

For schools interested in participating, additional permission was obtained from school boards and/or superintendents as necessary. Passive parental consent and student assent procedures were approved by the Yale School of Medicine Institutional Review Board and data collection sites. Parents were mailed letters detailing the study and notified to contact the school should they wish to decline their children’s participation. All procedures were performed in accordance with the Declaration of Helsinki and its amendments. Answers were anonymous and confidential, and students were reminded that their participation was voluntary. The refusal rate was low (<1%).

### 2.2. Measures

The surveys were administered at each school in a single day. The survey consisted of 154 questions that evaluated a range of characteristics such as socio-demographic information, health correlates, including substance use and other risk behaviors, and gambling measures. The survey included validated measures such as the Massachusetts Gambling Screen (MAGS) [35], and novel items which, although not validated, have been previously used [19,32,36,37,38,39,40], including in other youth surveys such as the Youth Risk Behavior Surveillance [41,42].

#### 2.2.1. Socio-Demographics

Socio-demographic variables included age, gender, race/ethnicity, grade level in school, and family structure (e.g., living with one parent).

#### 2.2.2. Health Correlates

Adolescents responded to questions investigating involvement in extracurricular activities (yes/no); grade average (A’s and B’s, mostly C’s, D’s or lower); lifetime tobacco smoking (never, occasionally, regularly), marijuana use (yes/no), other drug use (yes/no), and alcohol use (yes/no); past 30-days alcohol use (never, light, moderate, heavy) and caffeine use (none, 1–2 per day, 3+ per day); past 30-days weapon-carrying (gun, knife, or club) (yes/no), not going to school because of feeling unsafe (yes/no) and being threatened by a weapon (yes/no); past 12-months involvement in a physical fight (yes/no) and getting injured in a physical fight requiring the treatment of a doctor or nurse (yes/no). Adolescents were queried about bodyweight, which was categorized as underweight (body mass index [BMI] ≤ 18.5 kg/m^2^), ‘normal’ weight (18.6–24.9 kg/m^2^) and overweight/obese (BMI ≥ 25 kg/m^2^). Lastly, depression/dysphoria was assessed dichotomously (yes/no) by asking “During the past 12 months, did you ever feel so sad or hopeless almost every day for two weeks or more in a row that you stopped doing some usual activities”?

#### 2.2.3. Gambling Perceptions

Adolescents responded to questions investigating the perceived importance (important vs. not important) of gambling-prevention approaches. Participants were also queried on perceptions of their parents’ attitudes towards their gambling (disapprove/neither approve nor disapprove/approve) and whether they had any concerns about the gambling behavior of a family member (yes/no).

#### 2.2.4. Problem-Gambling Severity/Gambling Behaviors

Problem-gambling severity was assessed using the MAGS, a self-report instrument based on DSM-IV criteria for pathological gambling that has shown adequate reliability and predictive and construct validity [35]. Participants were also assessed on the presence (yes/no) of multiple gambling behaviors including locations (online, school, casino); triggers (pressure, anxiety); motivations (excitement, financial, escape, social); partners (family, friends, adults, siblings, strangers, alone); and age of onset (≤8, 9–11, 12–14 and ≥15 years old). Participants were also questioned about gambling types, which were categorized as strategic (gambling on cards or making bets on video or arcade games, dice, pool, or other games of skill; bets with bookies), non-strategic (instant lottery or scratch tickets; participating in bingo) and machine (poker or other electronic gambling machines) gambling.

### 2.3. Procedures

Of the 4523 adolescents who participated in the survey, 2556 students provided an answer to the question “Have you ever experienced a growing tension or anxiety that can only be relieved by shopping?” Students who answered “yes” (*n* = 373, 14.59%) were classified as having negative-reinforcement shopping, and those who answered “no” (*n* = 2183, 85.41%) were classified as not having negative-reinforcement shopping. Of these 2556 students, 2252 provided dichotomous (yes/no) information on past-year gambling and were included in gambling-related analyses.

Similar to procedures described in previous studies [19,32,36,37,38,39,40], we stratified adolescents who acknowledged gambling in the past year into two problem-gambling-severity groups based on the endorsement of at least one DSM-IV pathological gambling (PG) criterion as assessed by the MAGS (at-risk/problem gambling [ARPG] versus low-risk gambling). Likewise, similar to procedures described in previous studies [37,39], we also used the MAGS to stratify adolescents into DSM-IV PG and DSM-5 gambling-disorder (GD) groups. Separate PG and GD groups were created to account for the fact that DSM-5 dropped the illegal acts criterion that was present in DSM-IV. Adolescents who reported gambling and acknowledged at least five and at least four (excluding illegal acts criterion) criteria were classified as having PG and GD, respectively.

### 2.4. Statistical Analysis

Data were double-entered and randomly spot-checked to maintain accuracy. Between-group differences in socio-demographics, health correlates, gambling perceptions and problem-gambling severity/gambling behaviors between groups (with negative-reinforcement shopping versus without) were examined using Chi-square tests. Bonferroni correction for multiple comparisons was applied such that *p*-values of *p* < 0.0017 were considered significant. In addition, binomial and multinomial logistic regressions models were constructed to measure the magnitude of the observed associations through odds ratio (OR) with a 95% confidence interval (CI). All regression models included sociodemographic characteristics (age, gender, race/ethnicity, grade level in school, and family structure) as covariates to adjust for potential confounding effects. Significance for regression models was set at *p* < 0.05. Statistical analyses were conducted with IBM SPSS 27.

## 3. Results

### 3.1. Socio-Demographics

The frequencies and chi-square results of socio-demographics stratified by negative-reinforcement-shopping status are described in Table 1. A significant between-group difference was observed for gender (χ^2^ = 38.01, *p* < 0.001), with a larger proportion of females in the negative-reinforcement-shopping group (82.1% vs. 65.9%), and for ethnicity (χ^2^ = 15.80, *p* < 0.001), with a larger proportion of Hispanic youth in the negative-reinforcement-shopping group (20.9% vs. 13%).

### 3.2. Health Correlates

The frequencies and chi-square results and odds ratios of health correlates stratified by negative-reinforcement-shopping status are described in Table A1 (Appendix A) and Table 2, respectively. Negative-reinforcement shopping was associated with lifetime occasional (OR = 2.13; 95% CI = 1.57, 2.88; *p* < 0.001) and regular (OR = 2.52; 95% CI = 1.70, 3.73; *p* < 0.001) smoking; lifetime use of alcohol (OR = 2.59; 95% CI = 1.41, 4.76; *p* = 0.002), marijuana (OR = 2.26; 95% CI = 1.71, 2.99; *p* < 0.001), and other drugs (OR = 2.35; 95% CI = 1.52, 3.65; *p* < 0.001); moderate alcohol drinking (OR = 1.59; 95% CI = 1.04, 2.43; *p* = 0.03); drinking three or more cups of caffeine per day (OR = 1.58; 95% CI = 1.02, 2.42; *p* = 0.04); dysphoria/depression (OR = 1.72; 95% CI = 1.29, 2.29; *p* < 0.001); weapon-carrying (OR = 1.59; 95% CI = 1.07, 2.37; *p* = 0.02); feeling unsafe at school (OR = 3.61; 95% CI = 2.41, 5.41; *p* < 0.001); having been threatened with a weapon (OR = 2.12; 95% CI = 1.51, 2.98; *p* < 0.001); and having been injured in a physical fight requiring treatment by a doctor or nurse (OR = 3.62; 95% CI = 2.36, 5.57; *p* < 0.001).

### 3.3. Gambling Perceptions

The frequencies and chi-square results of gambling perceptions stratified by negative-reinforcement-shopping status are described in Table 3. A larger proportion of youth with negative-reinforcement shopping relative to those without classified as unimportant the following gambling prevention approaches: participating in activities that are fun and free of gambling (χ^2^ = 22.69, *p* < 0.001; 23.1% vs. 13.3%); fear of losing valuable possessions, close friends, and relatives (χ^2^ = 15.03, *p* < 0.001; 14.7% vs. 8.2%); and learning about the risks of gambling from parents (χ^2^ = 11.28, *p* < 0.001; 21.8% vs. 14.7%).

### 3.4. Problem-Gambling Severity/Gambling Behaviors

The frequencies and chi-square results and odds ratios of problem-gambling severity/gambling behaviors stratified by negative-reinforcement-shopping status are described in Table A2 (Appendix A) and Table 4, respectively. Negative-reinforcement shopping was associated with problem-gambling severity and disordered-gambling status—ARPG (OR = 1.74; 95% CI = 1.08, 2.82; *p* = 0.02), PG (OR = 3.76; 95% CI = 1.70, 8.28; *p* = 0.001), and GD (OR = 3.5; 95% CI = 1.66, 7.39; *p* = 0.001); non-strategic gambling (OR = 1.41; 95% CI = 1.02, 1.96; *p* = 0.04); gambling in a casino (OR = 3.00; 95% CI = 1.77, 5.09; *p* < 0.001); experiencing anxiety prior to gambling (OR = 3.19; 95% CI = 1.30, 7.82; *p* = 0.01); earlier age at onset of gambling—9–11 years (OR = 0.15; 95% CI = 0.06, 0.38; *p* < 0.001), 12–14 years (OR = 0.43; 95% CI = 0.24, 0.79; *p* = 0.01), and ≥15 years (OR = 0.41; 95% CI = 0.20, 0.82; *p* = 0.01); gambling alone (OR = 2.18, 95% CI = 1.10, 4.35; *p* = 0.03) and with strangers (OR = 2.29; 95% CI = 1.10, 4.73; *p* = 0.03).

## 4. Discussion

The present study investigated, in an exploratory fashion, the socio-demographics, health correlates, gambling perceptions and problem-gambling severity/gambling behaviors associated with negative-reinforcement shopping in a large sample of high-school students in Connecticut. We found that individuals who acknowledged experiencing a growing tension or anxiety that could only be relieved by shopping (i.e., had negative-reinforcement shopping) were more likely to be female and Hispanic. We also found that those individuals were more likely to report lifetime (e.g., tobacco, alcohol, and other drugs) and current substance use (e.g., alcohol), dysphoria/depression, and having experienced violence (e.g., weapon-carrying, having been threatened by a weapon, been involved in serious fights with resultant injuries, and felt unsafe due to violence). Additionally, we found that negative-reinforcement shopping was partially linked to more permissive gambling attitudes as reflected by, for example, classifying learning about gambling risks from parents as unimportant. Lastly, we found that negative-reinforcement shopping was associated with problematic gambling behavior, anxiety as a trigger for gambling, non-strategic gambling, gambling in casinos, and gambling alone or with strangers. Taken together, these findings highlight adolescent negative-reinforcement shopping to be linked to multiple adverse measures of health and functioning and gambling beliefs and behaviors.

### 4.1. Socio-Demographics

Our finding of girls being more likely to endorse negative-reinforcement shopping is in line with research on gender-related differences in coping strategies. Coping refers to the cognitive and behavioral processes adopted to manage external and/or internal demands that are perceived as exceeding the person’s usual resources [43,44,45]. Although there are multiple coping strategies [46], theoretical models [43,44,47,48] have often described two different coping mechanisms. More specifically, problem-focused coping has been used to describe actions that are undertaken to eliminate the stressor, whereas emotion-focused coping has been used to describe actions that are performed with the intention of eliminating the emotions elicited by the stressor. Males and females have been shown to differ in the coping strategies they typically adopt, as the latter tend to use more emotion-focused strategies [49,50], which may explain the greater proportion of girls who engaged in shopping to relieve tension/anxiety. However, it is also possible that due to differences in sociocultural norms, females may be more likely to report shopping-related concerns, a possibility that has been previously suggested for PS [51]. Regardless, the finding underscores the importance of considering gender in the identification of negative-reinforcement shopping.

We also found that Hispanic youth were more likely to report negative-reinforcement shopping. To the best of our knowledge, this association has not been previously described and suggests the importance of considering ethnicity when considering shopping behaviors, as with other behaviors. For example, research on food marketing has suggested that marketing for Hispanic individuals may be less likely to promote healthy eating, which may contribute to obesity in Latinos [52], and it is possible that similar ethnic differences in marketing practices could facilitate the adoption of shopping to alleviate stressors among this ethnic group, although we note that this possibility is currently speculative and requires direct examination.

### 4.2. Health and Functioning

Our hypothesis that negative-reinforcement shopping would be associated with substance use, dysphoria/depression, and violence-related measures was confirmed by our results. Previous research has indicated individuals who adopt emotion-focused coping strategies are more likely to experience higher levels of psychopathology, including depression, substance use and aggressive/violent behaviors [53,54,55]. Similar associations have also been described for PS in adolescents [19]. Prevention and therapeutic strategies for substance use and depression may impact engagement with negative-reinforcement shopping, and additional research should directly examine this problem.

### 4.3. Gambling Perceptions

Our hypothesis that individuals with negative-reinforcement shopping would display more permissive attitudes towards gambling was partially confirmed. In line with our hypothesis, we found that a larger proportion of individuals with negative-reinforcement shopping classified learning about the risks of gambling with their parents as unimportant, which could indicate the presence of communication barriers between parents and youth with negative-reinforcement shopping. Decreased communication between parents and children may result in increased problem-gambling severity, and effective parental communication may contribute to decreased problems arising from gambling and other risk-taking behaviors, e.g., alcohol use [56,57]. In this way, interventions aimed at improving parent–youth communication could help prevent problematic risk behaviors among adolescents with negative-reinforcement shopping. We also found that a larger proportion of individuals with negative-reinforcement shopping classified participating in activities free of gambling as unimportant, which could indicate that these individuals may benefit from the active promotion of gambling-free leisure activities. Nonetheless, we note that direct examination of these possibilities is required.

However, contrary to our hypothesis, we did not observe a relationship with perceived parental permissiveness towards gambling. Thus, our findings are in line with previous research which has demonstrated associations between perceived parental permissiveness and positive reinforcement motivations (e.g., sensation/excitement-seeking) and related constructs such as impulsivity [14,58,59], and problem-focused rather than emotion-focused coping strategies [60]. Although our study did not evaluate parenting styles in depth [61,62], some of the items in our survey evaluated the perceived importance of parental control over their children’s gambling behavior (e.g., parent/guardian strictness about gambling, parent/guardian not permitting card games for money at home), and we found non-significant differences after applying Bonferroni correction for multiple comparisons.

### 4.4. Problem-Gambling Severity/Gambling Behaviors

Our hypothesis that negative-reinforcement shopping would be associated with ARPG and GD was confirmed. Clinically, previous research has demonstrated that the frequencies of PS and GD among treatment-seeking individuals with the other condition are higher than the prevalence estimates of each condition in the community [63,64,65]. Our group previously reported no significant association between at-risk/PS and ARPG in the high-school-survey sample, and PG and GD had not been investigated [32]. The findings in our present and past [32] studies suggest that negative-reinforcement motivations to shop may be linked more closely to problem-gambling behaviors than some other features of PS. More generally, the findings could be indicative of the potential promise of considering dimensional mechanisms spanning different categories of addictive behaviors, rather than merely the categories themselves. This rationale is in line with large-scale initiatives such as the National Institute of Mental Health Research Domain Criteria [66] and the Hierarchical Taxonomy of Psychopathology [67]. Clinically, the findings highlight the importance of negative-reinforcement motivations for shopping and suggest that assessment with a single question assessing this construct may aid in identifying at-risk youth.

We hypothesize that the identification of at-risk youth could be improved by considering reward-based motivations for engagement with short-term rewarding behaviors (e.g., shopping, gambling, video-gaming, etc.) because individuals may engage in several such behaviors for similar reasons. Our findings regarding gambling behaviors may be partially supportive of this hypothesis. Although we did not find a direct association between negative-reinforcement shopping and gambling to escape/relieve dysphoria, youth with negative-reinforcement shopping were more likely to report anxiety as a trigger for gambling. Additionally, individuals who gamble as an emotion-focused coping strategy may be more likely to prefer non-strategic forms of gambling because of a dissociative-like attention-absorbing state (‘dark flow’) [68]. Dissociation may also facilitate impairment of control over financial limits [69], which could be a partial explanation for why youth with negative-reinforcement shopping were less likely to classify the fear of losing valuables as a gambling prevention strategy as important. Gambling in casinos may facilitate the occurrence of dissociative experiences [70], and adolescents who gamble at casinos have been found to endorse the desire to escape from dysphoria as a motivation to gamble [71]. Nonetheless, we note that these possibilities are currently speculative and require direct examination. It is possible that the associations between negative-reinforcement shopping and ARPG, PG and GD observed in this study could be explained by other factors, e.g., ones related to materialism [72]. Regardless, because adolescents are particularly susceptible to ARPG [73,74] and ARPG has been associated with negative health correlates in adolescence [37,75] and later in early adulthood [75,76], additional research aimed at better identifying adolescent ARPG is currently warranted and in line with public health needs and initiatives [77]. Our study provides new findings that could foster future research on the matter.

### 4.5. Strengths and Limitations

Despite our large sample size and the representativeness of our sample, our study has limitations that should be noted. Only 56% of the surveyed adolescents provided an answer to the question querying about negative-reinforcement shopping. It is possible that individuals who did not provide answers may show weaker/stronger associations between negative-reinforcement shopping and socio-demographics, health correlates, gambling perceptions, and problem-gambling severity/gambling behaviors. However, most sociodemographic variables assessed did not show differences between individuals completing versus not completing the negative-reinforcement shopping measure with the exception of male gender. However, sociodemographic variables (including gender) were included as covariates in all regression models to adjust for their potentially confounding effects. The number of youths with negative-reinforcement shopping was relatively small which precluded further examination of health correlates and gambling behaviors stratified by problem-gambling severity. It is possible, for example, that the negative-reinforcement-shopping status mediates stronger/weaker associations between ARPG and health correlates or gambling behaviors, but we were unable to address this question. Second, we used self-reported answers to only one question to classify individuals as having (or not) negative-reinforcement shopping. Future studies should consider a more detailed examination of underlying motivations to engage in shopping. Nonetheless, our study is helpful in demonstrating that a simple question might be helpful in identifying at-risk youth, although the potential utility and validity of screening adolescents in this manner also requires direct examination by future research. Third, our study was cross-sectional and consequently, we are not capable of inferring on longitudinal patterns of the associations observed in this study. Fourth, data were collected approximately 15 years ago, and while they may provide an important historical comparator, more recent studies are warranted to examine negative-reinforcement shopping and its correlates in a current environment. This may be particularly important given the ease of internet-based shopping and other potentially risky behaviors such as gambling now as compared to 15 years ago.

## 5. Conclusions

This study evaluated the socio-demographics, health correlates, gambling perceptions, and problem-gambling severity/gambling behaviors of adolescents who acknowledged engaging in shopping to relieve tension or anxiety. We found that youth with negative-reinforcement shopping were more likely to be female and Hispanic; report the use of alcohol, tobacco, marijuana, and other drugs; describe more permissive attitudes towards gambling; meet criteria for GD; gamble in casinos, alone and with strangers; experience dysphoria/depression; and acknowledge aggressive/violent behaviors such as weapon-carrying and physical fighting. Taken together, these findings highlight adolescent negative-reinforcement shopping as being linked to multiple youth risk behaviors. Additional research aimed at replicating and extending the current findings is warranted. Likewise, additional efforts to identify and prevent negative outcomes among these at-risk adolescents are warranted. The findings suggest that certain groups (e.g., girls and Hispanic youth) may be particularly vulnerable to experiencing negative-reinforcement shopping and may be particularly important to consider in efforts targeting this behavior. The finding of negative-reinforcement mechanisms motivating engagement in potentially addictive behaviors in females more so than males is consistent with the wider literature [78]. The current findings also resonate with a recent study examining negative-reinforcement gambling in which minority group status (including being Hispanic, Black or Asian-American) was linked to this construct, and the construct was linked to multiple adverse measures of health and functioning [79]. Thus, future studies and interventions should consider how escaping from negative mood states such as anxiety or tension may lead to engagement in potentially addictive behaviors, how minority youth may be particularly vulnerable, and how interventions may be developed to help youth find alternate, more healthy coping strategies. How such interventions may be developed, tested and implemented in academic settings also warrants direct examination.

## Figures and Tables

**Table 1:** Socio-demographic characteristics stratified by negative-reinforcement-shopping status.

	Non-Negative-Reinforcement		Negative-Reinforcement			
	N	%	N	%	χ^2^	*p*
Gender					**38.01**	**<0.001**
Male	738	34.1	66	17.9		
Female	1425	65.9	302	82.1		
Race/Ethnicity						
Caucasian/White					1.16	0.28
No	509	23.5	96	26.1		
Yes	1657	76.5	272	73.9		
African American/Black					0.63	0.43
No	1937	89.4	324	88		
Yes	229	10.6	44	12		
Asian American					1.56	0.21
No	2084	96.2	349	94.8		
Yes	82	3.8	19	5.2		
Hispanic					**15.8**	**<0.001**
No	1830	87	283	79.1		
Yes	274	13	75	20.9		
Other					3.21	0.07
No	1850	85.4	301	81.8		
Yes	316	14.6	67	18.2		
Grade					0.39	0.94
9th	678	31.1	119	32.1		
10th	606	27.8	98	26.4		
11th	566	26	99	26.7		
12th	327	15	55	14.8		
Age					1.19	0.55
≤14 years	320	18.5	63	21.2		
15–17 years	1211	70.2	202	68		
≥18 years	195	11.3	32	10.8		
Family structure					3.27	0.2
One parent	490	22.8	95	26		
Two parents	1547	72	247	67.5		
Other	112	5.2	24	6.6		

Bold indicates significant findings.

**Table 2:** Health correlates stratified by negative-reinforcement-shopping status (odds ratio).

	Negative-Reinforcement vs. Non-Negative-Reinforcement		
Variable	OR	95% CI	*p*
*Academic and extracurricular*			
Any extracurricular activities	1.03	0.74, 1.42	0.88
Grade average ^1^			
Mostly C’s	1.18	0.87, 1.60	0.28
D’s or lower	1.38	0.87, 2.18	0.17
*Substance use*			
Smoking, ever ^2^			
Occasionally	**2.13**	**1.57, 2.88**	**<0.001**
Regularly	**2.52**	**1.70, 3.73**	**<0.001**
Marijuana use, ever	**2.26**	**1.71, 2.99**	**<0.001**
Other drug use, ever	**2.35**	**1.52, 3.65**	**<0.001**
Alcohol use, ever	**2.59**	**1.41, 4.76**	**0.002**
Alcohol use, current ^3^			
Light	1.50	0.99, 2.28	0.06
Moderate	**1.59**	**1.04, 2.43**	**0.03**
Heavy	1.36	0.75, 2.44	0.31
Caffeine use ^4^			
1, 2 per day	1.07	0.71, 1.60	0.76
3+ per day	**1.58**	**1.02, 2.42**	**0.04**
*Mood*			
Dysphoria/Depression	**1.72**	**1.29, 2.29**	**<0.001**
*Weight*			
Body mass index ^5^			
Underweight	1.22	0.83, 1.80	0.32
Overweight/Obese	0.79	0.52, 1.18	0.25
*Violence*			
Carrying a weapon	**1.59**	**1.07, 2.37**	**0.02**
Felt unsafe	**3.61**	**2.41, 5.41**	**<0.001**
Threatened by weapon	**2.12**	**1.51, 2.98**	**<0.001**
Physical fighting	1.27	0.95, 1.71	0.11
Physical fighting with injury	**3.62**	**2.35, 5.57**	**<0.001**

Note: all models were adjusted for age, gender, ethnicity, grade, and family structure. Bold indicates significant findings. Abbreviations: OR = odds ratio; ^1^ Ref: A’s and B’s; ^2^ Ref: Never; ^3^ Ref: Never regular; ^4^ Ref: None; ^5^ Ref: ‘Normal’.

**Table 3:** Gambling perceptions stratified by negative-reinforcement-shopping status.

	Non-Negative Reinforcement		Negative Reinforcement			
Variable	N	%	N	%	χ^2^	*p*
Parent perception of gambling					12.11	0.002
Disapprove	991	51.2	161	49.8		
Neither approve nor disapprove	852	44	131	40.6		
Approve	94	4.9	31	9.6		
Family concern					8.21	0.004
No	1784	88	280	82.4		
Yes	244	12	60	17.6		
*Perceived importance for preventing gambling problems in teens*						
Checking identification for purchasing lottery tickets					5.61	0.02
Not important	292	14.1	66	18.9		
Important	1786	85.9	283	81.1		
Hanging out with friends who don’t gamble					5.26	0.02
Not important	486	23.5	101	29.3		
Important	1578	76.5	244	70.7		
Participating in activities that are fun and free of gambling					**22.69**	**<0.001**
Not important	275	13.3	80	23.1		
Important	1790	86.7	266	76.9		
Fear of losing valuable possessions, close friends, and relatives					**15.03**	**<0.001**
Not important	169	8.2	51	14.7		
Important	1889	91.8	296	85.3		
Advertisements of the problems associated with gambling					1.23	0.27
Not important	398	19.4	75	22		
Important	1652	80.6	266	78		
Not having access to internet gambling at home					1.36	0.24
Not important	672	32.7	124	35.9		
Important	1380	67.3	221	64.1		
Parent/Guardian strictness about gambling					4.18	0.04
Not important	353	17.2	75	21.7		
Important	1701	82.8	270	78.3		
Warning from adults in family					4.17	0.04
Not important	331	16.1	71	20.6		
Important	1720	83.9	274	79.4		
Warning from, or listening to, peers					4.3	0.04
Not important	322	15.7	69	20.2		
Important	1729	84.3	273	79.8		
Having parents who don’t gamble					4.57	0.03
Not important	343	16.7	74	21.4		
Important	1707	83.3	271	78.6		
Learning about the risks of gambling in school					6.44	0.01
Not important	394	19.2	86	25.1		
Important	1657	80.8	256	74.9		
Learning about the risks of gambling from parents					**11.28**	**<0.001**
Not important	301	14.7	75	21.8		
Important	1749	85.3	269	78.2		
Learning about the risks of gambling from peers					2.1	0.15
Not important	376	18.3	74	21.6		
Important	1675	81.7	268	78.4		
Adults not involving kids in gambling					3.03	0.08
Not important	286	14	60	17.5		
Important	1762	86	282	82.5		
Parent/Guardian not allowing card games (for money) at home					7.37	0.007
Not important	637	31.1	132	38.5		
Important	1412	68.9	211	61.5		

Bold indicates significant findings.

**Table 4:** Problem-gambling severity/gambling behaviors stratified by negative-reinforcement status (odds ratio).

	Negative-Reinforcement vs. Non-Negative-Reinforcement		
Variable	OR	95% CI	*p*
*Problem-gambling severity*			
At-risk/problem gambling	**1.74**	**1.08, 2.82**	**0.02**
Pathological gambling	**3.76**	**1.70, 8.28**	**0.001**
Gambling disorder	**3.50**	**1.66, 7.39**	**0.001**
Gambling type			
Machine gambling	1.00	0.68, 1.47	0.99
Strategic gambling	1.04	0.61, 1.77	0.88
Non-strategic gambling	**1.41**	**1.02–1.96**	**0.04**
Gambling location			
Internet	1.45	0.92, 2.27	0.11
School	1.40	0.93, 2.10	0.1
Casino	**3.00**	**1.77, 5.09**	**<0.001**
Gambling motivation			
Excitement/fun	1.08	0.79, 1.49	0.62
Financial	1.20	0.85, 1.68	0.3
Escape/relieve dysphoria	1.38	0.94, 2.03	0.1
Social	1.14	0.76, 1.69	0.53
Gambling urges			
Pressure	1.81	0.96, 3.41	0.07
Anxiety	**3.19**	**1.30, 7.82**	**0.01**
Age of onset ^1^			
9–11 years	**0.15**	**0.06, 0.38**	**<0.001**
12–14 years	**0.43**	**0.24, 0.79**	**0.006**
≥15 years	**0.41**	**0.20, 0.82**	**0.01**
Gambling partners			
Alone	**2.18**	**1.10, 4.35**	**0.03**
Friends	1.06	0.77, 1.45	0.74
Parents	1.40	0.90, 2.18	0.14
Other adults	1.67	0.94, 2.96	0.08
Family	1.13	0.78, 1.63	0.52
Strangers	**2.29**	**1.10, 4.73**	**0.03**
Siblings	1.25	0.82, 1.91	0.3

Note: all models were adjusted for age, gender, ethnicity, grade, and family structure. Bold indicates significant findings; Abbreviations: OR = odds ratio; ^1^ Ref: ≤ 8 years.

## Data Availability

The data from this study may be made available to researchers upon reasonable request while honoring agreements made with participating schools and considering ongoing analyses. The data are not publicly available due to ethical restrictions.

## Appendix A

**Table A1:** Health correlates stratified by negative-reinforcement-shopping status (frequencies).

	Non-Negative Reinforcement		Negative Reinforcement			
Variable	N	%	N	%	χ^2^	*p*
*Academic and extracurricular*						
Any extracurricular activities					0.05	0.82
No	474	21.7	83	22.3		
Yes	1709	78.3	290	77.7		
Grade average					6.11	0.05
A’s and B’s	1303	61.2	201	56.3		
Mostly C’s	640	30	111	31.1		
D’s or lower	187	8.8	45	12.6		
*Substance use*						
Smoking, ever					**53.45**	**<0.001**
Never	1337	62.7	155	43.3		
Occasionally	550	25.8	125	34.9		
Regularly	246	11.5	78	21.8		
Marijuana use, ever					**46.56**	**<0.001**
No	1288	62.5	151	43.1		
Yes	773	37.5	199	56.9		
Alcohol use, ever					**15.29**	**<0.001**
No	244	11.7	17	4.8		
Yes	1846	88.3	340	95.2		
Alcohol use, current					3.94	0.27
Never regular	414	30.1	65	24.2		
Light	397	28.9	82	30.5		
Moderate	403	29.3	88	32.7		
Heavy	162	11.8	34	12.6		
Other drug use, ever					**20.41**	**<0.001**
No	1666	91.4	251	83.1		
Yes	156	8.6	51	16.9		
Caffeine use					10.52	0.005
None	345	16.1	51	13.9		
1–2 per day	1211	56.6	185	50.5		
3+ per day	583	27.3	130	35.5		
*Mood*						
Dysphoria/depression					**46.08**	**<0.001**
No	1654	79	226	62.6		
Yes	440	21	135	37.4		
*Weight*						
Body mass index					3.66	0.16
Underweight	237	11.8	44	13.7		
Normal	1376	68.7	229	71.1		
Overweight/obese	390	19.5	49	15.2		
*Violence*						
Weapon-carrying					2.27	0.13
No	1782	82.8	292	79.6		
Yes	370	17.2	75	20.4		
Felt unsafe					**67.34**	**<0.001**
No	2024	94.3	302	82.1		
Yes	123	5.7	66	17.9		
Threatened by weapon					**23.4**	**<0.001**
No	1837	85.4	276	75.4		
Yes	313	14.6	90	24.6		
Physical fighting					3.15	0.08
No	1458	67.9	230	63.2		
Yes	689	32.1	134	36.8		
Physical fighting with injury					**56.58**	**<0.001**
No	2017	94	302	82.7		
Yes	128	6	63	17.3		

Bold indicates statistical significance.

**Table A2:** Problem-gambling severity/gambling behaviors stratified by negative-reinforcement-shopping status (frequencies).

	Non-Negative Reinforcement		Negative Reinforcement			
Variable	N	%	N	%	χ^2^	*p*
*Problem-gambling severity*						
At-risk/problem gambling					5.78	0.02
No	679	69.5	94	59.9		
Yes	298	30.5	63	40.1		
Pathological gambling					**42.79**	**<0.001**
No	935	95.7	129	82.2		
Yes	42	4.3	28	17.8		
Gambling disorder					**39.83**	**<0.001**
No	919	94.8	127	80.9		
Yes	50	5.2	30	19.1		
*Gambling type*						
Machine gambling					0.4	0.84
No	1595	83.3	270	82.8		
Yes	320	16.7	56	17.2		
Strategic gambling					1.64	0.2
No	132	6.9	29	8.8		
Yes	1790	93.1	299	91.2		
Non-strategic gambling					5.87	0.02
No	623	32.4	84	25.7		
Yes	1299	67.6	243	74.3		
*Gambling location*						
Internet					1.28	0.26
No	1698	88.9	280	86.7		
Yes	213	11.1	43	13.3		
School					0.49	0.48
No	1500	78.2	259	79.9		
Yes	418	21.8	65	20.1		
Casino					**34.18**	**<0.001**
No	1822	95.3	284	87.1		
Yes	89	4.7	42	12.9		
*Gambling motivation*						
Excitement/fun					2.46	0.12
No	1223	63.6	224	68.1		
Yes	700	36.4	105	31.9		
Financial					0.001	0.99
No	1386	72.1	237	72		
Yes	537	27.9	92	28		
Escape/relieve dysphoria					2.67	0.1
No	1629	84.7	267	81.2		
Yes	294	15.3	62	18.8		
Social					0.82	0.37
No	1543	80.2	271	82.4		
Yes	380	19.8	58	17.6		
*Gambling urges*						
Pressure					6.81	0.01
No	1822	95.8	294	92.5		
Yes	80	4.2	24	7.5		
Anxiety					**15.97**	**<0.001**
No	1060	96.5	161	89.9		
Yes	38	3.5	18	10.1		
*Early gambling*						
*Age of onset*					**21.63**	**<0.001**
≤8 years	99	12.3	35	26.3		
9–11 years	135	16.7	12	9		
12–14 years	310	38.4	51	38.3		
≥15 years	364	32.7	35	26.3		
*Gambling partners*						
Alone					4.49	0.03
No	1858	96.6	310	94.2		
Yes	65	3.4	19	5.8		
Friends					1.61	0.21
No	1204	62.6	218	66.3		
Yes	719	37.4	111	33.7		
Parents					1.73	0.19
No	1740	90.5	290	88.1		
Yes	183	9.5	39	11.9		
Other adults					2.75	0.1
No	1811	94.2	302	91.8		
Yes	112	5.8	27	8.2		
Family					0.003	0.96
No	1563	81.3	267	81.2		
Yes	360	18.7	62	18.8		
Strangers					8.27	0.004
No	1858	96.6	307	93.3		
Yes	65	3.4	22	6.7		
Siblings					1.17	0.28
No	1673	87	279	84.8		
Yes	250	13	50	15.2		

Bold indicates statistical significance.
